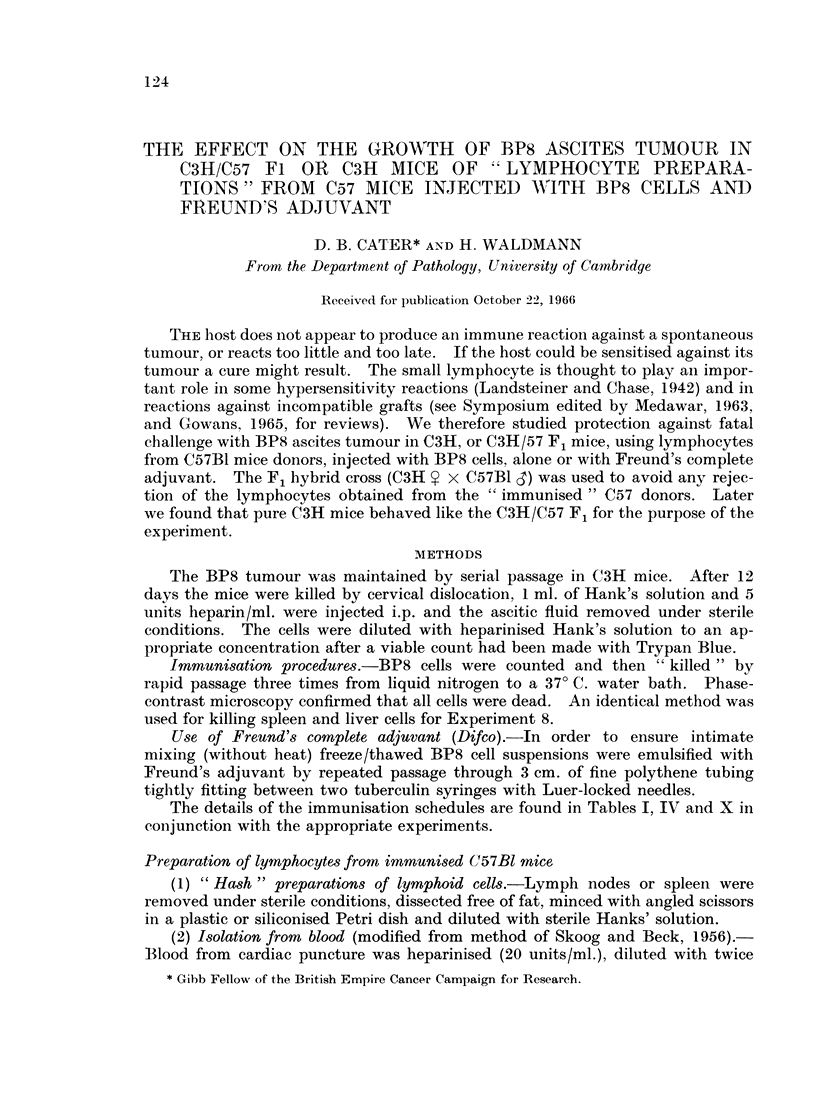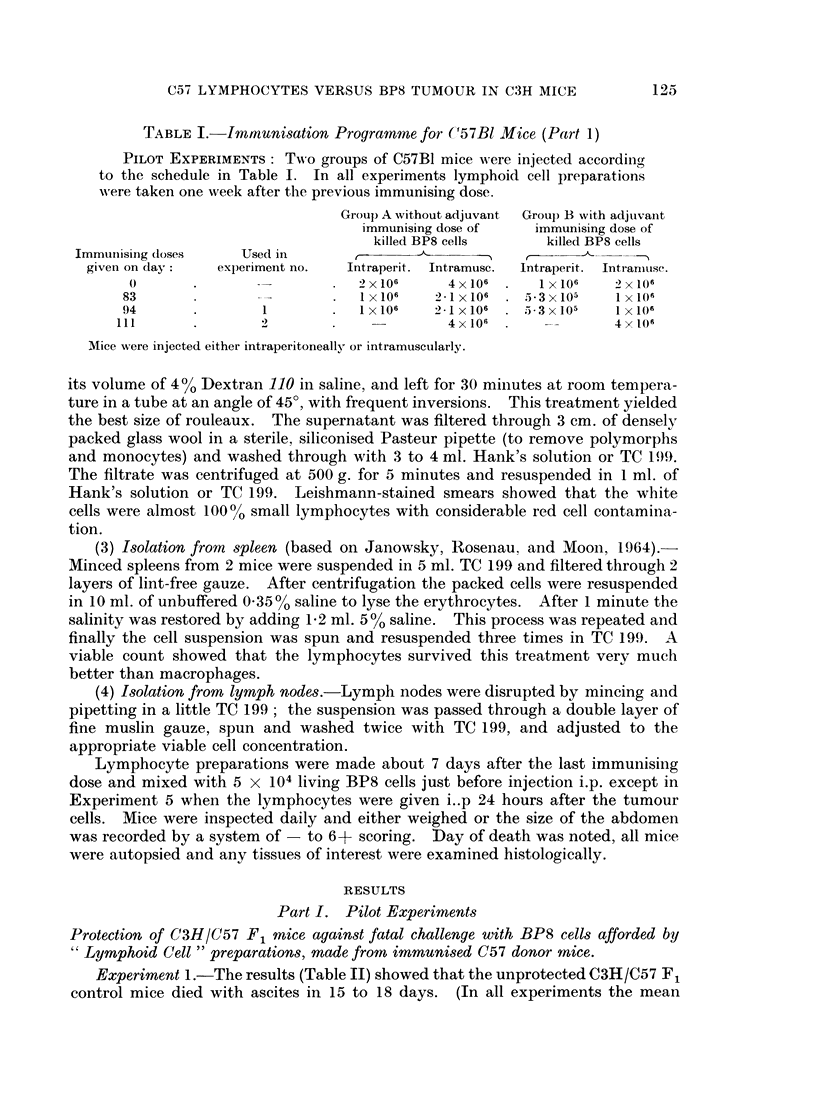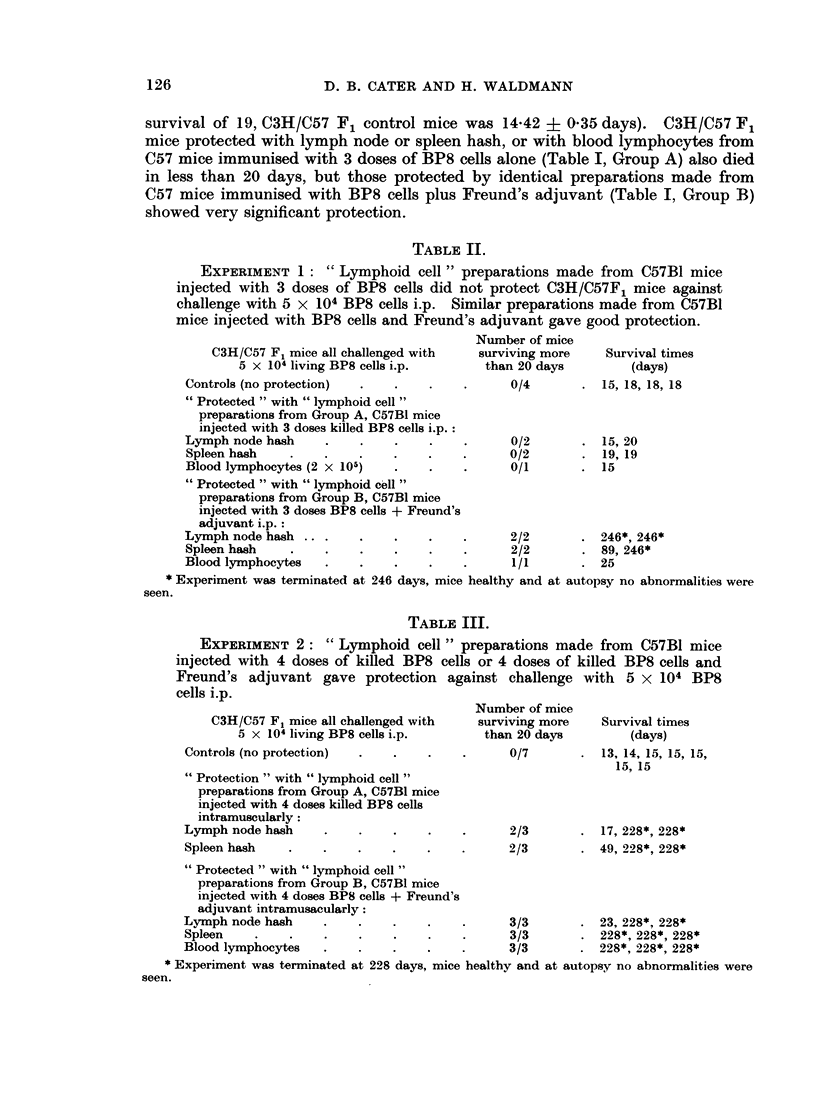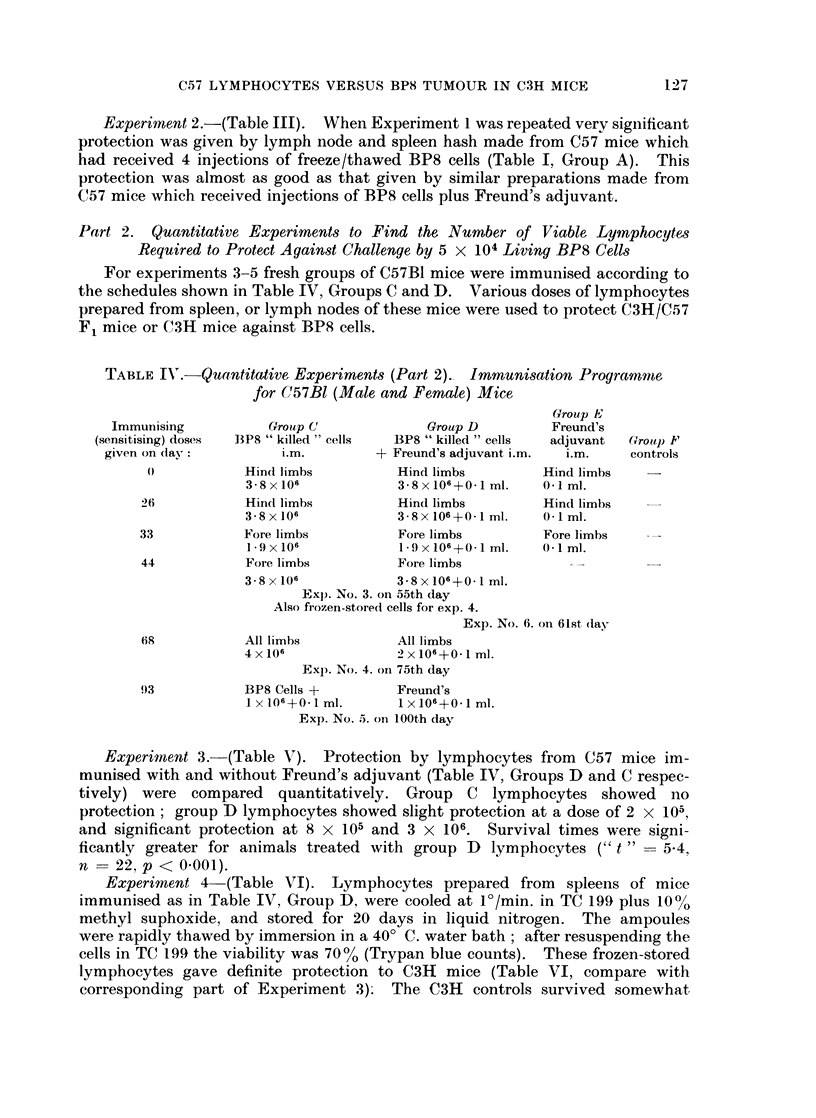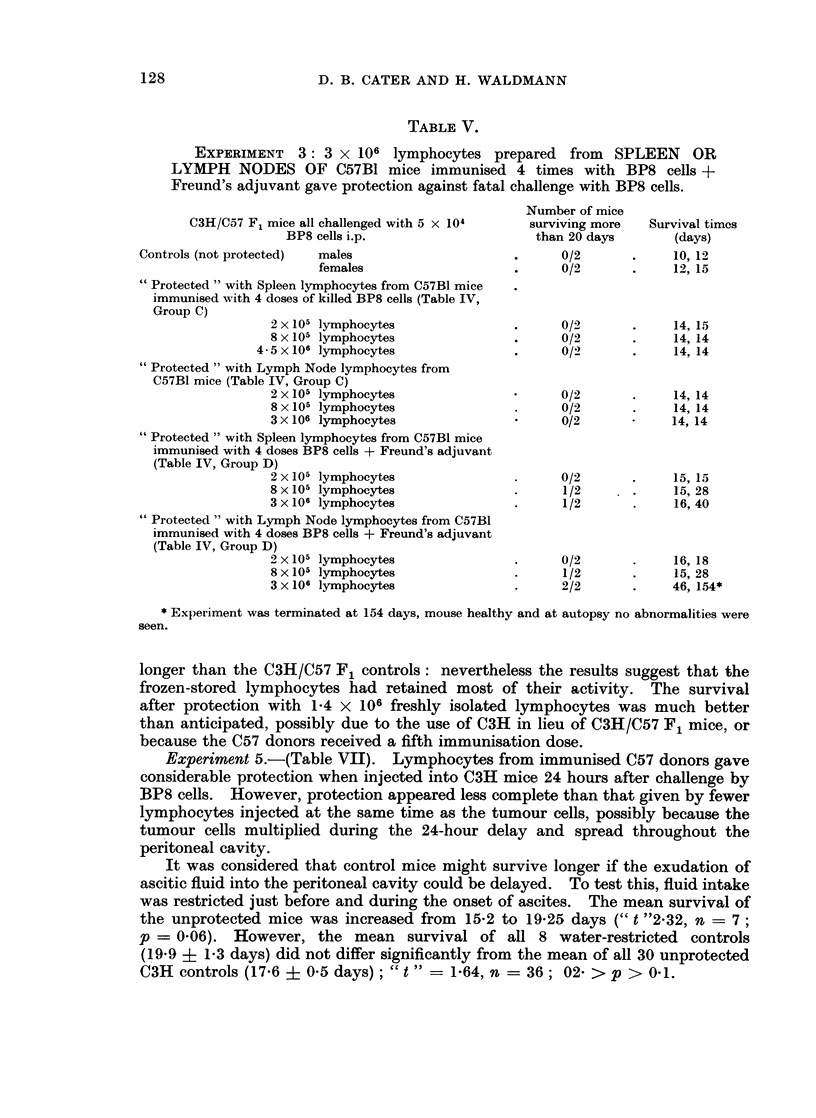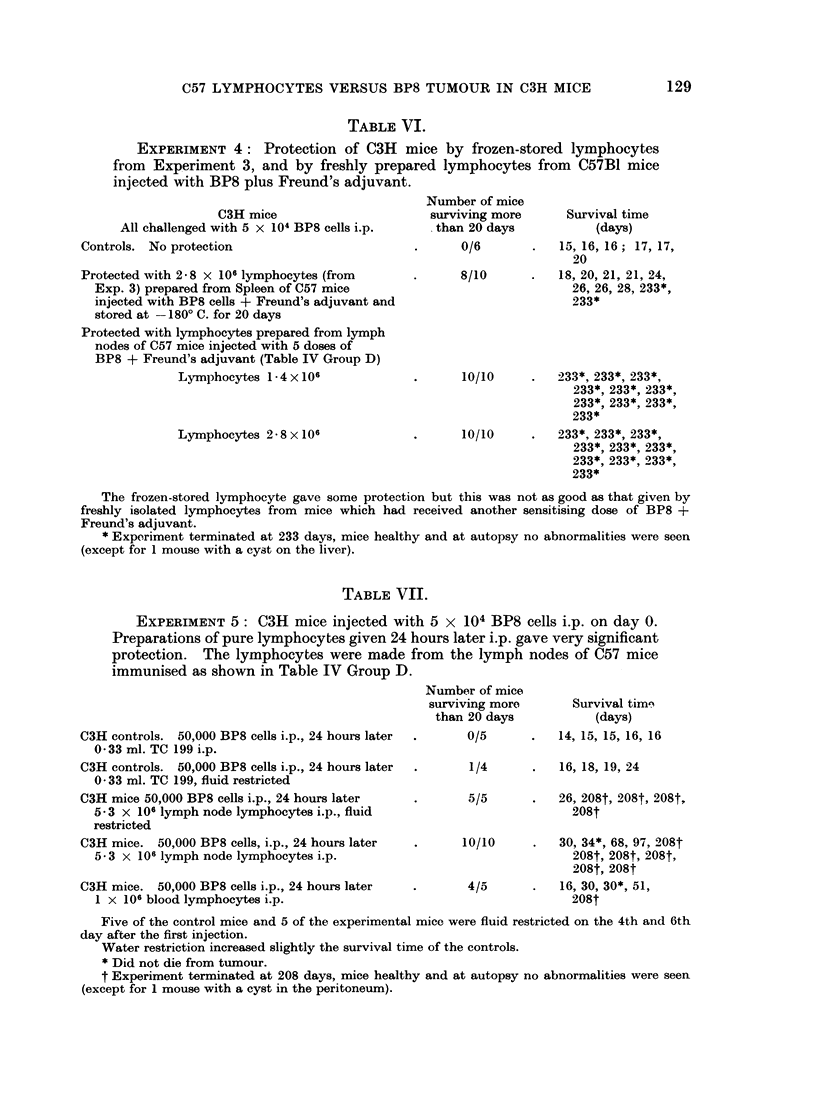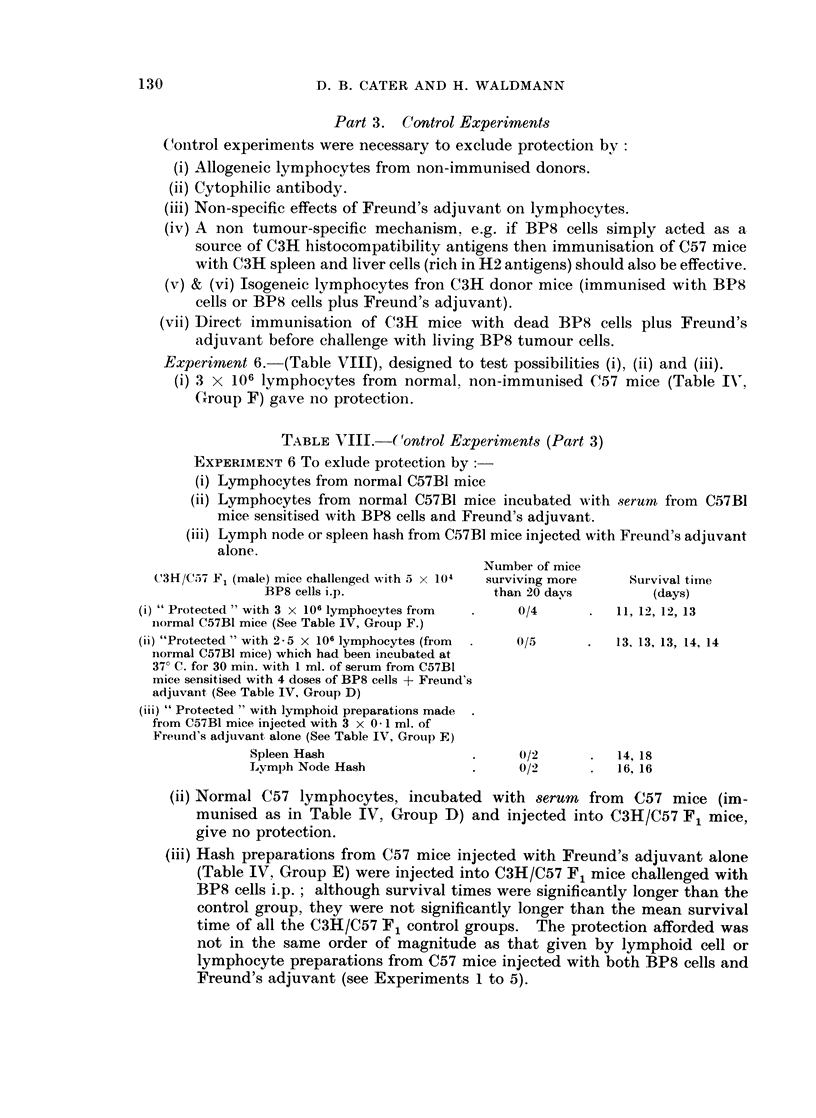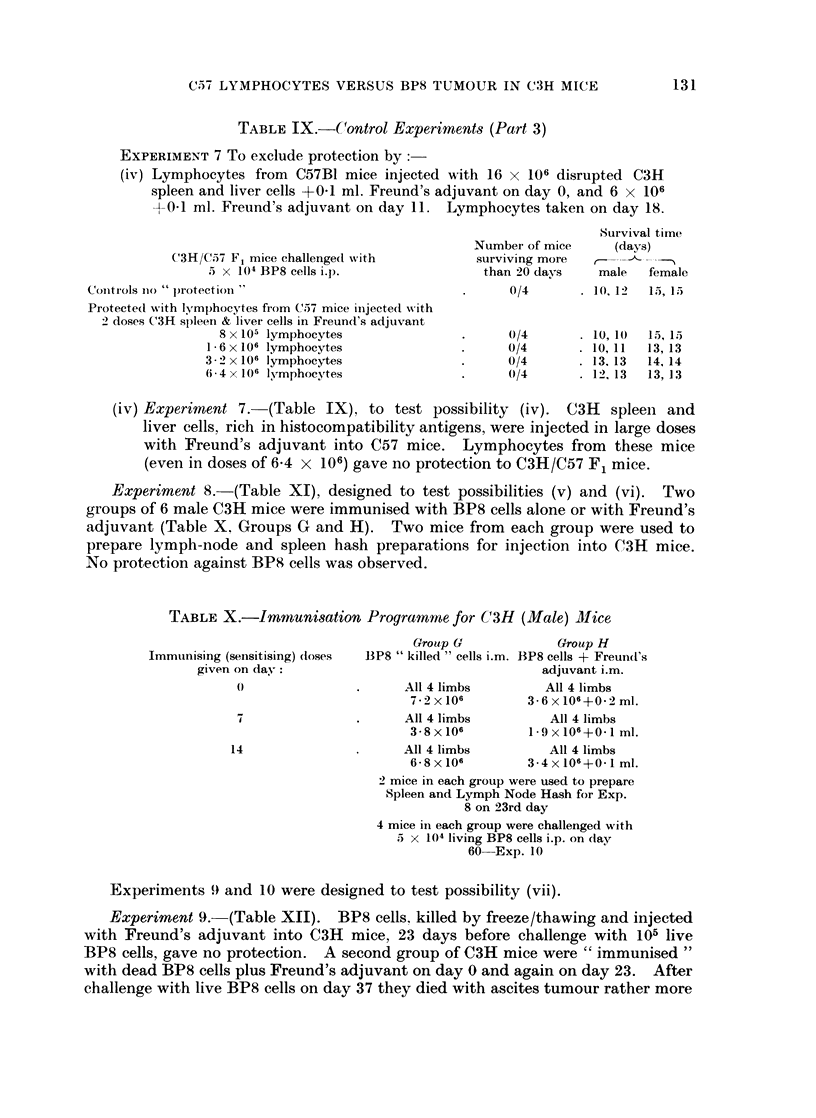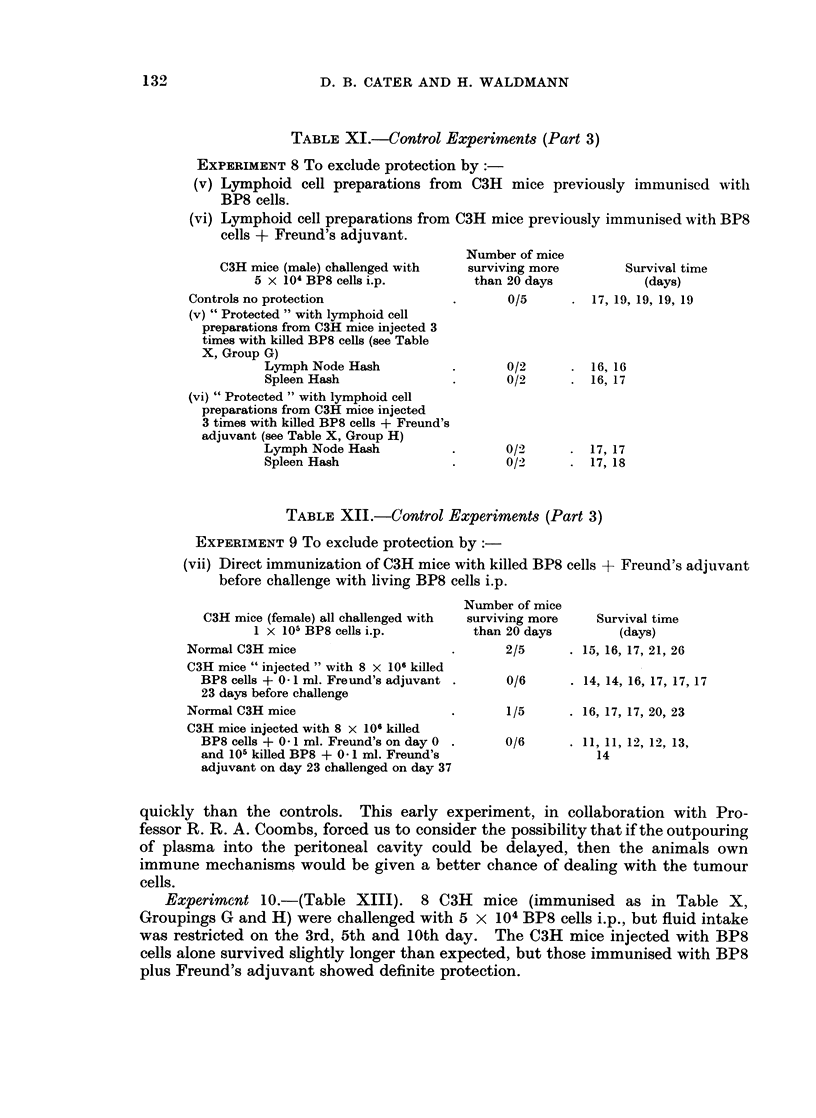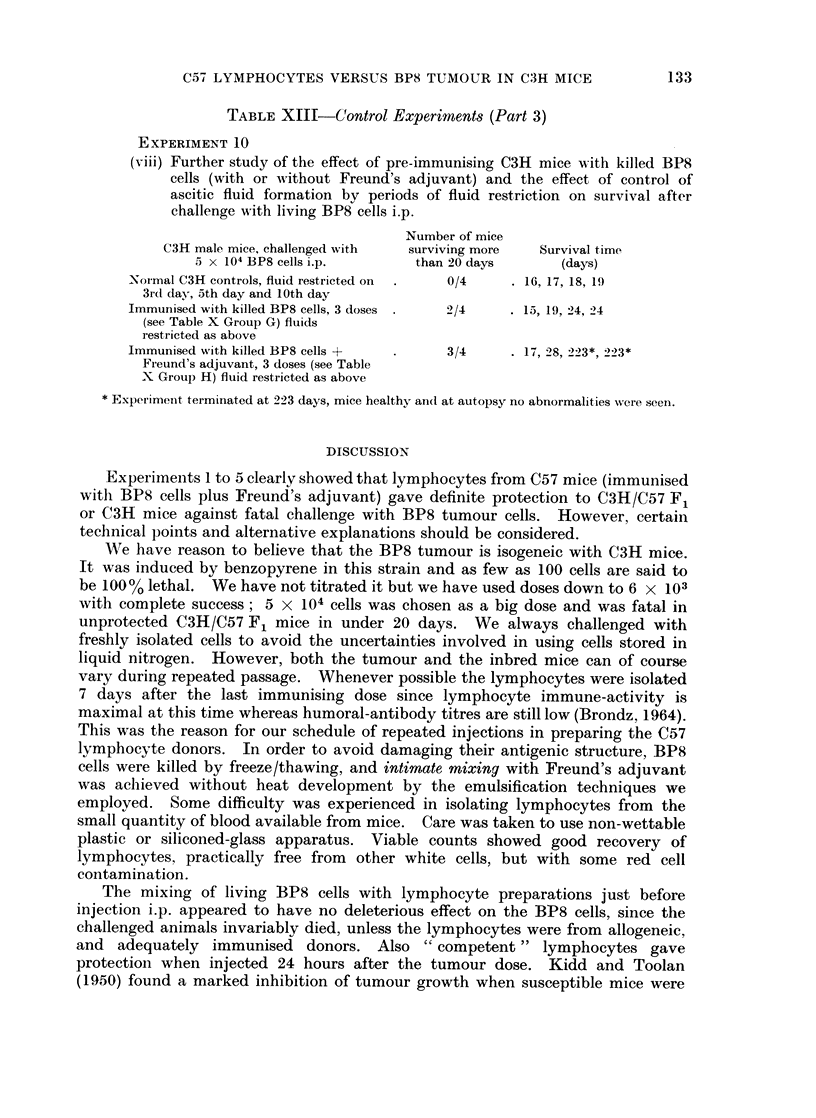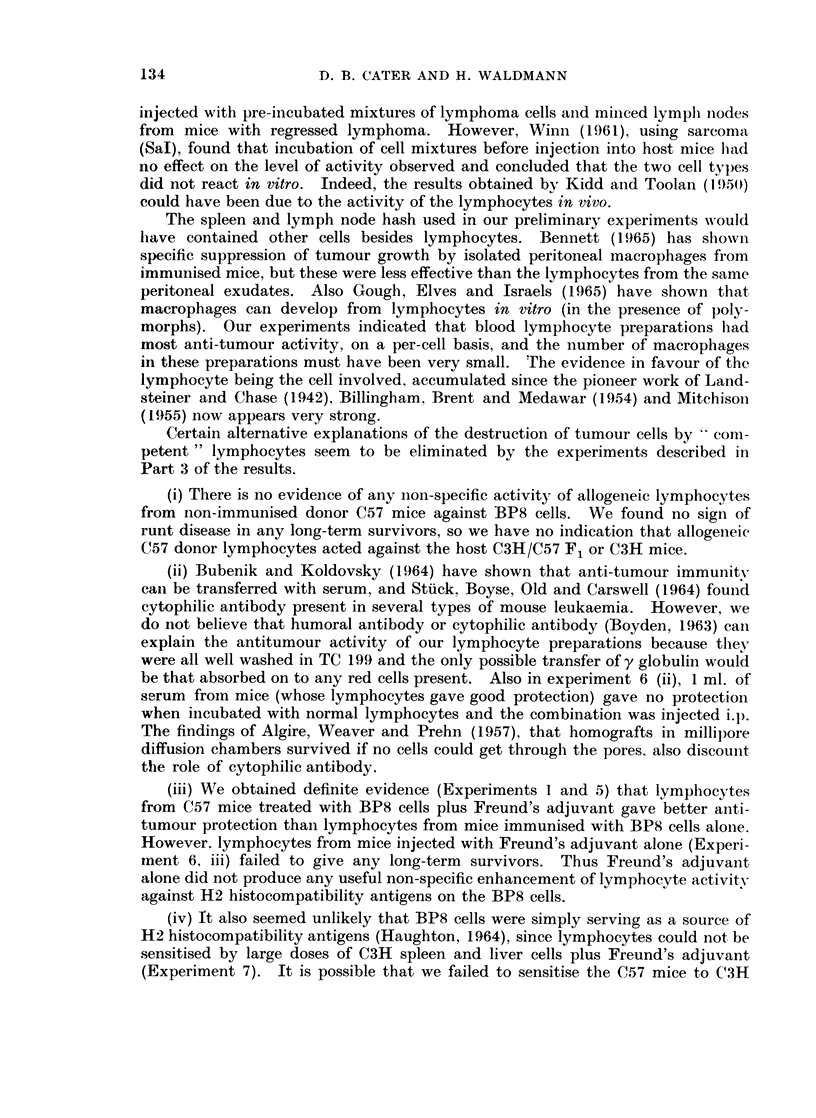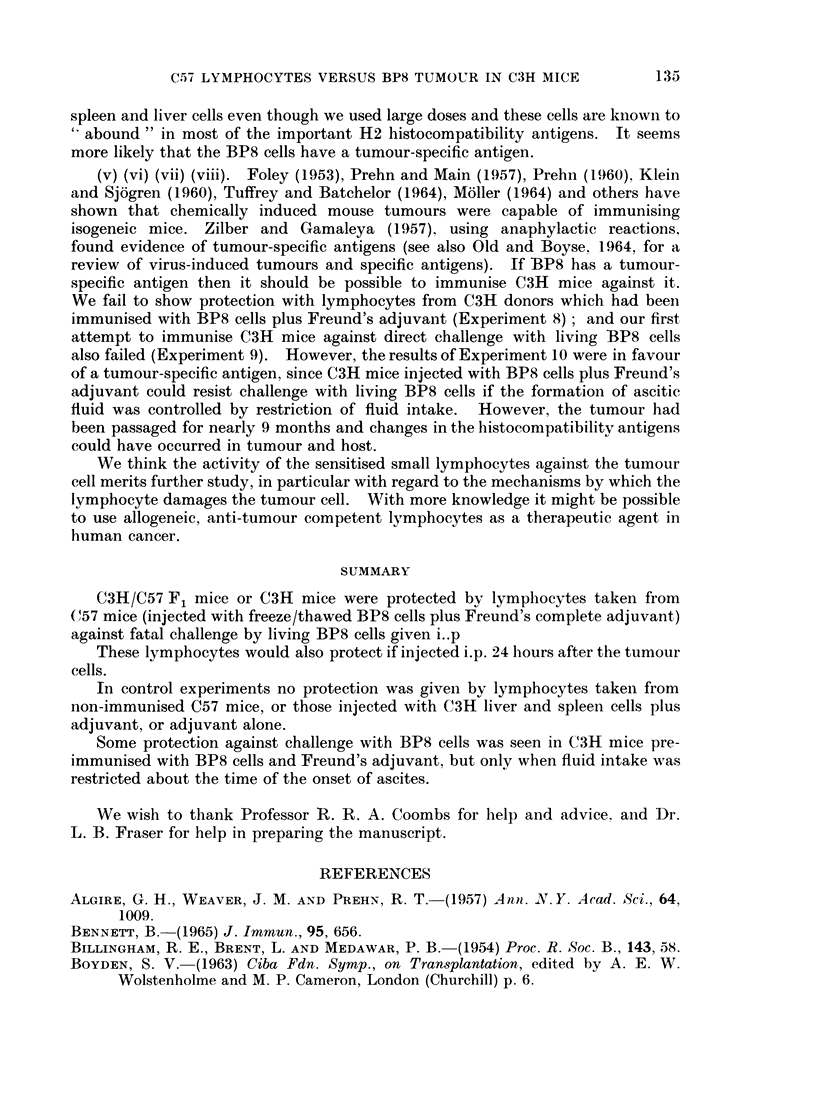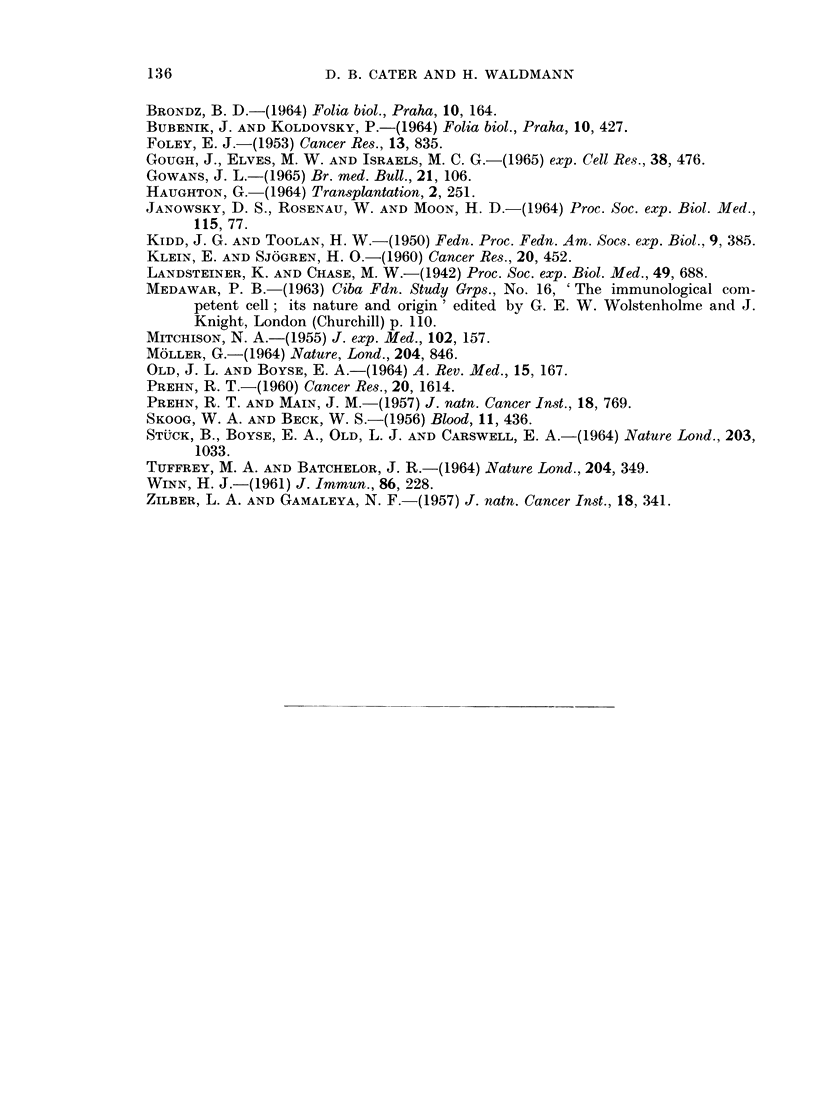# The effect on the growth of BP8 ascites tumour in C3H-C57 or C3H mice of "lymphocyte preparations" from C57 mice injected with BP8 cells and Freund's adjuvant.

**DOI:** 10.1038/bjc.1967.11

**Published:** 1967-03

**Authors:** D. B. Cater, H. Waldmann


### THE EFFECT ON THE GROWTH OF BP8 ASCITES TUMOUR IN C3H/C57 F1 OR C3H MICE OF "LYMPHOCYTE PREPARATIONS" FROM C57 MICE INJECTED WITH BP8 CELLS AND FREUND'S ADJUVANT

D. B. CATER* AND H. WALDMANN

*From the Department of Pathology, University of Cambridge*

Received for publication October 22, 1966

THE host does not appear to produce an immune reaction against a spontaneous tumour, or reacts too little and too late. If the host could be sensitised against its tumour a cure might result. The small lymphocyte is thought to play an important role in some hypersensitivity reactions (Landsteiner and Chase, 1942) and in reactions against incompatible grafts (see Symposium edited by Medawar, 1963, and Gowans, 1965, for reviews). We therefore studied protection against fatal challenge with BP8 ascites tumour in C3H, or C3H/57 $F_1$ mice, using lymphocytes from C57Bl mice donors, injected with BP8 cells, alone or with Freund's complete adjuvant. The $F_1$ hybrid cross (C3H ♀ × C57Bl ♂) was used to avoid any rejection of the lymphocytes obtained from the "immunised" C57 donors. Later we found that pure C3H mice behaved like the C3H/C57 $F_1$ for the purpose of the experiment.

### METHODS

The BP8 tumour was maintained by serial passage in C3H mice. After 12 days the mice were killed by cervical dislocation, 1 ml. of Hank's solution and 5 units heparin/ml. were injected i.p. and the ascitic fluid removed under sterile conditions. The cells were diluted with heparinised Hank's solution to an appropriate concentration after a viable count had been made with Trypan Blue.

*Immunisation procedures.*—BP8 cells were counted and then "killed" by rapid passage three times from liquid nitrogen to a 37° C. water bath. Phase-contrast microscopy confirmed that all cells were dead. An identical method was used for killing spleen and liver cells for Experiment 8.

*Use of Freund's complete adjuvant (Difco).*—In order to ensure intimate mixing (without heat) freeze/thawed BP8 cell suspensions were emulsified with Freund's adjuvant by repeated passage through 3 cm. of fine polythene tubing tightly fitting between two tuberculin syringes with Luer-locked needles.

The details of the immunisation schedules are found in Tables I, IV and X in conjunction with the appropriate experiments.

### *Preparation of lymphocytes from immunised C57Bl mice*

(1) *"Hash" preparations of lymphoid cells.*—Lymph nodes or spleen were removed under sterile conditions, dissected free of fat, minced with angled scissors in a plastic or siliconised Petri dish and diluted with sterile Hanks' solution.

(2) *Isolation from blood* (modified from method of Skoog and Beck, 1956).—Blood from cardiac puncture was heparinised (20 units/ml.), diluted with twice

* Gibb Fellow of the British Empire Cancer Campaign for Research.

TABLE I.—*Immunisation Programme for C57Bl Mice (Part 1)*

PILOT EXPERIMENTS : Two groups of C57Bl mice were injected according to the schedule in Table I. In all experiments lymphoid cell preparations were taken one week after the previous immunising dose.

| Immunising doses given on day : | Used in experiment no. | Group A without adjuvant immunising dose of killed BP8 cells: Intraperit. | Group A: Intramusc. | Group B with adjuvant immunising dose of killed BP8 cells: Intraperit. | Group B: Intramusc. |
|---|---|---|---|---|---|
| 0 | — | $2\times10^{6}$ | $4\times10^{6}$ | $1\times10^{6}$ | $2\times10^{6}$ |
| 83 | — | $1\times10^{6}$ | $2{\cdot}1\times10^{6}$ | $5{\cdot}3\times10^{5}$ | $1\times10^{6}$ |
| 94 | 1 | $1\times10^{6}$ | $2{\cdot}1\times10^{6}$ | $5{\cdot}3\times10^{5}$ | $1\times10^{6}$ |
| 111 | 2 | — | $4\times10^{6}$ | — | $4\times10^{6}$ |

Mice were injected either intraperitoneally or intramuscularly.

its volume of 4% Dextran *110* in saline, and left for 30 minutes at room temperature in a tube at an angle of 45°, with frequent inversions. This treatment yielded the best size of rouleaux. The supernatant was filtered through 3 cm. of densely packed glass wool in a sterile, siliconised Pasteur pipette (to remove polymorphs and monocytes) and washed through with 3 to 4 ml. Hank's solution or TC 199. The filtrate was centrifuged at 500 g. for 5 minutes and resuspended in 1 ml. of Hank's solution or TC 199. Leishmann-stained smears showed that the white cells were almost 100% small lymphocytes with considerable red cell contamination.

(3) *Isolation from spleen* (based on Janowsky, Rosenau, and Moon, 1964).—Minced spleens from 2 mice were suspended in 5 ml. TC 199 and filtered through 2 layers of lint-free gauze. After centrifugation the packed cells were resuspended in 10 ml. of unbuffered 0·35% saline to lyse the erythrocytes. After 1 minute the salinity was restored by adding 1·2 ml. 5% saline. This process was repeated and finally the cell suspension was spun and resuspended three times in TC 199. A viable count showed that the lymphocytes survived this treatment very much better than macrophages.

(4) *Isolation from lymph nodes.*—Lymph nodes were disrupted by mincing and pipetting in a little TC 199 ; the suspension was passed through a double layer of fine muslin gauze, spun and washed twice with TC 199, and adjusted to the appropriate viable cell concentration.

Lymphocyte preparations were made about 7 days after the last immunising dose and mixed with $5 \times 10^{4}$ living BP8 cells just before injection i.p. except in Experiment 5 when the lymphocytes were given i..p 24 hours after the tumour cells. Mice were inspected daily and either weighed or the size of the abdomen was recorded by a system of — to 6+ scoring. Day of death was noted, all mice were autopsied and any tissues of interest were examined histologically.

### RESULTS

### *Part I. Pilot Experiments*

*Protection of C3H/C57 $F_1$ mice against fatal challenge with BP8 cells afforded by "Lymphoid Cell" preparations, made from immunised C57 donor mice.*

*Experiment* 1.—The results (Table II) showed that the unprotected C3H/C57 $F_1$ control mice died with ascites in 15 to 18 days. (In all experiments the mean survival of 19, C3H/C57 $F_1$ control mice was $14{\cdot}42 \pm 0{\cdot}35$ days). C3H/C57 $F_1$ mice protected with lymph node or spleen hash, or with blood lymphocytes from C57 mice immunised with 3 doses of BP8 cells alone (Table I, Group A) also died in less than 20 days, but those protected by identical preparations made from C57 mice immunised with BP8 cells plus Freund's adjuvant (Table I, Group B) showed very significant protection.

TABLE II.

EXPERIMENT 1 : " Lymphoid cell " preparations made from C57Bl mice injected with 3 doses of BP8 cells did not protect C3H/C57$F_1$ mice against challenge with $5 \times 10^4$ BP8 cells i.p. Similar preparations made from C57Bl mice injected with BP8 cells and Freund's adjuvant gave good protection.

| C3H/C57 $F_1$ mice all challenged with $5 \times 10^4$ living BP8 cells i.p. | Number of mice surviving more than 20 days | Survival times (days) |
|---|---|---|
| Controls (no protection) . . . . | 0/4 | . 15, 18, 18, 18 |
| " Protected " with " lymphoid cell " preparations from Group A, C57Bl mice injected with 3 doses killed BP8 cells i.p. : | | |
| Lymph node hash . . . . . . | 0/2 | . 15, 20 |
| Spleen hash . . . . . . . | 0/2 | . 19, 19 |
| Blood lymphocytes ($2 \times 10^5$) . . . | 0/1 | . 15 |
| " Protected " with " lymphoid cell " preparations from Group B, C57Bl mice injected with 3 doses BP8 cells + Freund's adjuvant i.p. : | | |
| Lymph node hash .. . . . . . . | 2/2 | . 246*, 246* |
| Spleen hash . . . . . . . | 2/2 | . 89, 246* |
| Blood lymphocytes . . . . . . | 1/1 | . 25 |

* Experiment was terminated at 246 days, mice healthy and at autopsy no abnormalities were seen.

TABLE III.

EXPERIMENT 2 : " Lymphoid cell " preparations made from C57Bl mice injected with 4 doses of killed BP8 cells or 4 doses of killed BP8 cells and Freund's adjuvant gave protection against challenge with $5 \times 10^4$ BP8 cells i.p.

| C3H/C57 $F_1$ mice all challenged with $5 \times 10^4$ living BP8 cells i.p. | Number of mice surviving more than 20 days | Survival times (days) |
|---|---|---|
| Controls (no protection) . . . . | 0/7 | . 13, 14, 15, 15, 15, 15, 15 |
| " Protection " with " lymphoid cell " preparations from Group A, C57Bl mice injected with 4 doses killed BP8 cells intramuscularly : | | |
| Lymph node hash . . . . . . | 2/3 | . 17, 228*, 228* |
| Spleen hash . . . . . . . | 2/3 | . 49, 228*, 228* |
| " Protected " with " lymphoid cell " preparations from Group B, C57Bl mice injected with 4 doses BP8 cells + Freund's adjuvant intramusacularly : | | |
| Lymph node hash . . . . . . | 3/3 | . 23, 228*, 228* |
| Spleen . . . . . . . . . | 3/3 | . 228*, 228*, 228* |
| Blood lymphocytes . . . . . . | 3/3 | . 228*, 228*, 228* |

* Experiment was terminated at 228 days, mice healthy and at autopsy no abnormalities were seen.

*Experiment* 2.—(Table III). When Experiment 1 was repeated very significant protection was given by lymph node and spleen hash made from C57 mice which had received 4 injections of freeze/thawed BP8 cells (Table I, Group A). This protection was almost as good as that given by similar preparations made from C57 mice which received injections of BP8 cells plus Freund's adjuvant.

### *Part* 2. *Quantitative Experiments to Find the Number of Viable Lymphocytes Required to Protect Against Challenge by* $5 \times 10^4$ *Living BP*8 *Cells*

For experiments 3–5 fresh groups of C57Bl mice were immunised according to the schedules shown in Table IV, Groups C and D. Various doses of lymphocytes prepared from spleen, or lymph nodes of these mice were used to protect C3H/C57 $F_1$ mice or C3H mice against BP8 cells.

TABLE IV.—*Quantitative Experiments* (*Part* 2). *Immunisation Programme for C*57*Bl* (*Male and Female*) *Mice*

| Immunising (sensitising) doses given on day : | *Group C* BP8 " killed " cells i.m. | *Group D* BP8 " killed " cells + Freund's adjuvant i.m. | *Group E* Freund's adjuvant i.m. | *Group F* controls |
|---|---|---|---|---|
| 0 | Hind limbs $3 \cdot 8 \times 10^6$ | Hind limbs $3 \cdot 8 \times 10^6 + 0 \cdot 1$ ml. | Hind limbs $0 \cdot 1$ ml. | — |
| 26 | Hind limbs $3 \cdot 8 \times 10^6$ | Hind limbs $3 \cdot 8 \times 10^6 + 0 \cdot 1$ ml. | Hind limbs $0 \cdot 1$ ml. | — |
| 33 | Fore limbs $1 \cdot 9 \times 10^6$ | Fore limbs $1 \cdot 9 \times 10^6 + 0 \cdot 1$ ml. | Fore limbs $0 \cdot 1$ ml. | — |
| 44 | Fore limbs $3 \cdot 8 \times 10^6$ | Fore limbs $3 \cdot 8 \times 10^6 + 0 \cdot 1$ ml. | — | — |
| | Exp. No. 3. on 55th day Also frozen-stored cells for exp. 4. | | | |
| | | | Exp. No. 6. on 61st day | |
| 68 | All limbs $4 \times 10^6$ | All limbs $2 \times 10^6 + 0 \cdot 1$ ml. | | |
| | Exp. No. 4. on 75th day | | | |
| 93 | BP8 Cells + $1 \times 10^6 + 0 \cdot 1$ ml. | Freund's $1 \times 10^6 + 0 \cdot 1$ ml. | | |
| | Exp. No. 5. on 100th day | | | |

*Experiment* 3.—(Table V). Protection by lymphocytes from C57 mice immunised with and without Freund's adjuvant (Table IV, Groups D and C respectively) were compared quantitatively. Group C lymphocytes showed no protection ; group D lymphocytes showed slight protection at a dose of $2 \times 10^5$, and significant protection at $8 \times 10^5$ and $3 \times 10^6$. Survival times were significantly greater for animals treated with group D lymphocytes (" $t$ " $= 5{\cdot}4$, $n = 22$, $p < 0{\cdot}001$).

*Experiment* 4—(Table VI). Lymphocytes prepared from spleens of mice immunised as in Table IV, Group D, were cooled at 1°/min. in TC 199 plus 10% methyl suphoxide, and stored for 20 days in liquid nitrogen. The ampoules were rapidly thawed by immersion in a 40° C. water bath ; after resuspending the cells in TC 199 the viability was 70% (Trypan blue counts). These frozen-stored lymphocytes gave definite protection to C3H mice (Table VI, compare with corresponding part of Experiment 3): The C3H controls survived somewhat

TABLE V.

EXPERIMENT 3 : $3 \times 10^6$ lymphocytes prepared from SPLEEN OR LYMPH NODES OF C57Bl mice immunised 4 times with BP8 cells + Freund's adjuvant gave protection against fatal challenge with BP8 cells.

| C3H/C57 $F_1$ mice all challenged with $5 \times 10^4$ BP8 cells i.p. | | Number of mice surviving more than 20 days | Survival times (days) |
|---|---|---|---|
| Controls (not protected) | males | 0/2 | 10, 12 |
| | females | 0/2 | 12, 15 |
| " Protected " with Spleen lymphocytes from C57Bl mice immunised with 4 doses of killed BP8 cells (Table IV, Group C) | | | |
| | $2 \times 10^5$ lymphocytes | 0/2 | 14, 15 |
| | $8 \times 10^5$ lymphocytes | 0/2 | 14, 14 |
| | $4 \cdot 5 \times 10^6$ lymphocytes | 0/2 | 14, 14 |
| " Protected " with Lymph Node lymphocytes from C57Bl mice (Table IV, Group C) | | | |
| | $2 \times 10^5$ lymphocytes | 0/2 | 14, 14 |
| | $8 \times 10^5$ lymphocytes | 0/2 | 14, 14 |
| | $3 \times 10^6$ lymphocytes | 0/2 | 14, 14 |
| " Protected " with Spleen lymphocytes from C57Bl mice immunised with 4 doses BP8 cells + Freund's adjuvant (Table IV, Group D) | | | |
| | $2 \times 10^5$ lymphocytes | 0/2 | 15, 15 |
| | $8 \times 10^5$ lymphocytes | 1/2 | 15, 28 |
| | $3 \times 10^6$ lymphocytes | 1/2 | 16, 40 |
| " Protected " with Lymph Node lymphocytes from C57Bl immunised with 4 doses BP8 cells + Freund's adjuvant (Table IV, Group D) | | | |
| | $2 \times 10^5$ lymphocytes | 0/2 | 16, 18 |
| | $8 \times 10^5$ lymphocytes | 1/2 | 15, 28 |
| | $3 \times 10^6$ lymphocytes | 2/2 | 46, 154* |

* Experiment was terminated at 154 days, mouse healthy and at autopsy no abnormalities were seen.

longer than the C3H/C57 $F_1$ controls : nevertheless the results suggest that the frozen-stored lymphocytes had retained most of their activity. The survival after protection with $1 \cdot 4 \times 10^6$ freshly isolated lymphocytes was much better than anticipated, possibly due to the use of C3H in lieu of C3H/C57 $F_1$ mice, or because the C57 donors received a fifth immunisation dose.

*Experiment* 5.—(Table VII). Lymphocytes from immunised C57 donors gave considerable protection when injected into C3H mice 24 hours after challenge by BP8 cells. However, protection appeared less complete than that given by fewer lymphocytes injected at the same time as the tumour cells, possibly because the tumour cells multiplied during the 24-hour delay and spread throughout the peritoneal cavity.

It was considered that control mice might survive longer if the exudation of ascitic fluid into the peritoneal cavity could be delayed. To test this, fluid intake was restricted just before and during the onset of ascites. The mean survival of the unprotected mice was increased from 15·2 to 19·25 days (" $t$ "2·32, $n = 7$ ; $p = 0 \cdot 06$). However, the mean survival of all 8 water-restricted controls ($19 \cdot 9 \pm 1 \cdot 3$ days) did not differ significantly from the mean of all 30 unprotected C3H controls ($17 \cdot 6 \pm 0 \cdot 5$ days) ; " $t$ " $= 1 \cdot 64$, $n = 36$ ; $02 \cdot > p > 0 \cdot 1$.

TABLE VI.

EXPERIMENT 4: Protection of C3H mice by frozen-stored lymphocytes from Experiment 3, and by freshly prepared lymphocytes from C57Bl mice injected with BP8 plus Freund's adjuvant.

| C3H mice All challenged with $5 \times 10^4$ BP8 cells i.p. | Number of mice surviving more than 20 days | Survival time (days) |
|---|---|---|
| Controls. No protection | 0/6 | 15, 16, 16; 17, 17, 20 |
| Protected with $2 \cdot 8 \times 10^6$ lymphocytes (from Exp. 3) prepared from Spleen of C57 mice injected with BP8 cells + Freund's adjuvant and stored at −180° C. for 20 days | 8/10 | 18, 20, 21, 21, 24, 26, 26, 28, 233*, 233* |
| Protected with lymphocytes prepared from lymph nodes of C57 mice injected with 5 doses of BP8 + Freund's adjuvant (Table IV Group D) | | |
| Lymphocytes $1 \cdot 4 \times 10^6$ | 10/10 | 233*, 233*, 233*, 233*, 233*, 233*, 233*, 233*, 233*, 233* |
| Lymphocytes $2 \cdot 8 \times 10^6$ | 10/10 | 233*, 233*, 233*, 233*, 233*, 233*, 233*, 233*, 233*, 233* |

The frozen-stored lymphocyte gave some protection but this was not as good as that given by freshly isolated lymphocytes from mice which had received another sensitising dose of BP8 + Freund's adjuvant.

* Experiment terminated at 233 days, mice healthy and at autopsy no abnormalities were seen (except for 1 mouse with a cyst on the liver).

TABLE VII.

EXPERIMENT 5: C3H mice injected with $5 \times 10^4$ BP8 cells i.p. on day 0. Preparations of pure lymphocytes given 24 hours later i.p. gave very significant protection. The lymphocytes were made from the lymph nodes of C57 mice immunised as shown in Table IV Group D.

| | Number of mice surviving more than 20 days | Survival time (days) |
|---|---|---|
| C3H controls. 50,000 BP8 cells i.p., 24 hours later 0·33 ml. TC 199 i.p. | 0/5 | 14, 15, 15, 16, 16 |
| C3H controls. 50,000 BP8 cells i.p., 24 hours later 0·33 ml. TC 199, fluid restricted | 1/4 | 16, 18, 19, 24 |
| C3H mice 50,000 BP8 cells i.p., 24 hours later $5 \cdot 3 \times 10^6$ lymph node lymphocytes i.p., fluid restricted | 5/5 | 26, 208†, 208†, 208†, 208† |
| C3H mice. 50,000 BP8 cells, i.p., 24 hours later $5 \cdot 3 \times 10^6$ lymph node lymphocytes i.p. | 10/10 | 30, 34*, 68, 97, 208† 208†, 208†, 208†, 208†, 208† |
| C3H mice. 50,000 BP8 cells i.p., 24 hours later $1 \times 10^6$ blood lymphocytes i.p. | 4/5 | 16, 30, 30*, 51, 208† |

Five of the control mice and 5 of the experimental mice were fluid restricted on the 4th and 6th day after the first injection.

Water restriction increased slightly the survival time of the controls.

* Did not die from tumour.

† Experiment terminated at 208 days, mice healthy and at autopsy no abnormalities were seen (except for 1 mouse with a cyst in the peritoneum).

*Part* 3. *Control Experiments*

Control experiments were necessary to exclude protection by :

(i) Allogeneic lymphocytes from non-immunised donors.

(ii) Cytophilic antibody.

(iii) Non-specific effects of Freund's adjuvant on lymphocytes.

(iv) A non tumour-specific mechanism, e.g. if BP8 cells simply acted as a source of C3H histocompatibility antigens then immunisation of C57 mice with C3H spleen and liver cells (rich in H2 antigens) should also be effective.

(v) & (vi) Isogeneic lymphocytes fron C3H donor mice (immunised with BP8 cells or BP8 cells plus Freund's adjuvant).

(vii) Direct immunisation of C3H mice with dead BP8 cells plus Freund's adjuvant before challenge with living BP8 tumour cells.

*Experiment* 6.—(Table VIII), designed to test possibilities (i), (ii) and (iii).

(i) 3 × $10^6$ lymphocytes from normal, non-immunised C57 mice (Table IV, Group F) gave no protection.

TABLE VIII.—*Control Experiments* (*Part* 3)

EXPERIMENT 6 To exlude protection by :—

(i) Lymphocytes from normal C57Bl mice

(ii) Lymphocytes from normal C57Bl mice incubated with *serum* from C57Bl mice sensitised with BP8 cells and Freund's adjuvant.

(iii) Lymph node or spleen hash from C57Bl mice injected with Freund's adjuvant alone.

| C3H/C57 $F_1$ (male) mice challenged with 5 × $10^4$ BP8 cells i.p. | Number of mice surviving more than 20 days | Survival time (days) |
|---|---|---|
| (i) " Protected " with 3 × $10^6$ lymphocytes from normal C57Bl mice (See Table IV, Group F.) | 0/4 | 11, 12, 12, 13 |
| (ii) "Protected " with 2·5 × $10^6$ lymphocytes (from normal C57Bl mice) which had been incubated at 37° C. for 30 min. with 1 ml. of serum from C57Bl mice sensitised with 4 doses of BP8 cells + Freund's adjuvant (See Table IV, Group D) | 0/5 | 13, 13, 13, 14, 14 |
| (iii) " Protected " with lymphoid preparations made from C57Bl mice injected with 3 × 0·1 ml. of Freund's adjuvant alone (See Table IV, Group E) | | |
| Spleen Hash | 0/2 | 14, 18 |
| Lymph Node Hash | 0/2 | 16, 16 |

(ii) Normal C57 lymphocytes, incubated with *serum* from C57 mice (immunised as in Table IV, Group D) and injected into C3H/C57 $F_1$ mice, give no protection.

(iii) Hash preparations from C57 mice injected with Freund's adjuvant alone (Table IV, Group E) were injected into C3H/C57 $F_1$ mice challenged with BP8 cells i.p. ; although survival times were significantly longer than the control group, they were not significantly longer than the mean survival time of all the C3H/C57 $F_1$ control groups. The protection afforded was not in the same order of magnitude as that given by lymphoid cell or lymphocyte preparations from C57 mice injected with both BP8 cells and Freund's adjuvant (see Experiments 1 to 5).

TABLE IX.—*Control Experiments* (*Part* 3)

EXPERIMENT 7 To exclude protection by :—

(iv) Lymphocytes from C57Bl mice injected with $16 \times 10^6$ disrupted C3H spleen and liver cells +0·1 ml. Freund's adjuvant on day 0, and $6 \times 10^6$ +0·1 ml. Freund's adjuvant on day 11. Lymphocytes taken on day 18.

| C3H/C57 $F_1$ mice challenged with $5 \times 10^4$ BP8 cells i.p. | Number of mice surviving more than 20 days | Survival time (days) male | Survival time (days) female |
|---|---|---|---|
| Controls no " protection " | 0/4 | 10, 12 | 15, 15 |
| Protected with lymphocytes from C57 mice injected with 2 doses C3H spleen & liver cells in Freund's adjuvant | | | |
| $8 \times 10^5$ lymphocytes | 0/4 | 10, 10 | 15, 15 |
| $1 \cdot 6 \times 10^6$ lymphocytes | 0/4 | 10, 11 | 13, 13 |
| $3 \cdot 2 \times 10^6$ lymphocytes | 0/4 | 13, 13 | 14, 14 |
| $6 \cdot 4 \times 10^6$ lymphocytes | 0/4 | 12, 13 | 13, 13 |

(iv) *Experiment* 7.—(Table IX), to test possibility (iv). C3H spleen and liver cells, rich in histocompatibility antigens, were injected in large doses with Freund's adjuvant into C57 mice. Lymphocytes from these mice (even in doses of $6{\cdot}4 \times 10^6$) gave no protection to C3H/C57 $F_1$ mice.

*Experiment* 8.—(Table XI), designed to test possibilities (v) and (vi). Two groups of 6 male C3H mice were immunised with BP8 cells alone or with Freund's adjuvant (Table X, Groups G and H). Two mice from each group were used to prepare lymph-node and spleen hash preparations for injection into C3H mice. No protection against BP8 cells was observed.

TABLE X.—*Immunisation Programme for C3H* (*Male*) *Mice*

| Immunising (sensitising) doses given on day : | *Group G* BP8 " killed " cells i.m. | *Group H* BP8 cells + Freund's adjuvant i.m. |
|---|---|---|
| 0 | All 4 limbs $7 \cdot 2 \times 10^6$ | All 4 limbs $3 \cdot 6 \times 10^6 + 0 \cdot 2$ ml. |
| 7 | All 4 limbs $3 \cdot 8 \times 10^6$ | All 4 limbs $1 \cdot 9 \times 10^6 + 0 \cdot 1$ ml. |
| 14 | All 4 limbs $6 \cdot 8 \times 10^6$ | All 4 limbs $3 \cdot 4 \times 10^6 + 0 \cdot 1$ ml. |

2 mice in each group were used to prepare Spleen and Lymph Node Hash for Exp. 8 on 23rd day

4 mice in each group were challenged with $5 \times 10^4$ living BP8 cells i.p. on day 60—Exp. 10

Experiments 9 and 10 were designed to test possibility (vii).

*Experiment* 9.—(Table XII). BP8 cells, killed by freeze/thawing and injected with Freund's adjuvant into C3H mice, 23 days before challenge with $10^5$ live BP8 cells, gave no protection. A second group of C3H mice were " immunised " with dead BP8 cells plus Freund's adjuvant on day 0 and again on day 23. After challenge with live BP8 cells on day 37 they died with ascites tumour rather more

TABLE XI.—*Control Experiments* (*Part* 3)

EXPERIMENT 8 To exclude protection by :—

(v) Lymphoid cell preparations from C3H mice previously immunised with BP8 cells.

(vi) Lymphoid cell preparations from C3H mice previously immunised with BP8 cells + Freund's adjuvant.

| C3H mice (male) challenged with $5 \times 10^4$ BP8 cells i.p. | Number of mice surviving more than 20 days | Survival time (days) |
|---|---|---|
| Controls no protection | 0/5 | 17, 19, 19, 19, 19 |
| (v) " Protected " with lymphoid cell preparations from C3H mice injected 3 times with killed BP8 cells (see Table X, Group G) | | |
| Lymph Node Hash | 0/2 | 16, 16 |
| Spleen Hash | 0/2 | 16, 17 |
| (vi) " Protected " with lymphoid cell preparations from C3H mice injected 3 times with killed BP8 cells + Freund's adjuvant (see Table X, Group H) | | |
| Lymph Node Hash | 0/2 | 17, 17 |
| Spleen Hash | 0/2 | 17, 18 |

TABLE XII.—*Control Experiments* (*Part* 3)

EXPERIMENT 9 To exclude protection by :—

(vii) Direct immunization of C3H mice with killed BP8 cells + Freund's adjuvant before challenge with living BP8 cells i.p.

| C3H mice (female) all challenged with $1 \times 10^5$ BP8 cells i.p. | Number of mice surviving more than 20 days | Survival time (days) |
|---|---|---|
| Normal C3H mice | 2/5 | 15, 16, 17, 21, 26 |
| C3H mice " injected " with $8 \times 10^6$ killed BP8 cells + 0·1 ml. Freund's adjuvant 23 days before challenge | 0/6 | 14, 14, 16, 17, 17, 17 |
| Normal C3H mice | 1/5 | 16, 17, 17, 20, 23 |
| C3H mice injected with $8 \times 10^6$ killed BP8 cells + 0·1 ml. Freund's on day 0 and $10^5$ killed BP8 + 0·1 ml. Freund's adjuvant on day 23 challenged on day 37 | 0/6 | 11, 11, 12, 12, 13, 14 |

quickly than the controls. This early experiment, in collaboration with Professor R. R. A. Coombs, forced us to consider the possibility that if the outpouring of plasma into the peritoneal cavity could be delayed, then the animals own immune mechanisms would be given a better chance of dealing with the tumour cells.

*Experiment* 10.—(Table XIII). 8 C3H mice (immunised as in Table X, Groupings G and H) were challenged with $5 \times 10^4$ BP8 cells i.p., but fluid intake was restricted on the 3rd, 5th and 10th day. The C3H mice injected with BP8 cells alone survived slightly longer than expected, but those immunised with BP8 plus Freund's adjuvant showed definite protection.

TABLE XIII—*Control Experiments* (*Part* 3)

EXPERIMENT 10

(viii) Further study of the effect of pre-immunising C3H mice with killed BP8 cells (with or without Freund's adjuvant) and the effect of control of ascitic fluid formation by periods of fluid restriction on survival after challenge with living BP8 cells i.p.

| C3H male mice, challenged with $5 \times 10^4$ BP8 cells i.p. | Number of mice surviving more than 20 days | Survival time (days) |
|---|---|---|
| Normal C3H controls, fluid restricted on 3rd day, 5th day and 10th day | 0/4 | 16, 17, 18, 19 |
| Immunised with killed BP8 cells, 3 doses (see Table X Group G) fluids restricted as above | 2/4 | 15, 19, 24, 24 |
| Immunised with killed BP8 cells + Freund's adjuvant, 3 doses (see Table X Group H) fluid restricted as above | 3/4 | 17, 28, 223*, 223* |

* Experiment terminated at 223 days, mice healthy and at autopsy no abnormalities were seen.

### DISCUSSION

Experiments 1 to 5 clearly showed that lymphocytes from C57 mice (immunised with BP8 cells plus Freund's adjuvant) gave definite protection to C3H/C57 $F_1$ or C3H mice against fatal challenge with BP8 tumour cells. However, certain technical points and alternative explanations should be considered.

We have reason to believe that the BP8 tumour is isogeneic with C3H mice. It was induced by benzopyrene in this strain and as few as 100 cells are said to be 100% lethal. We have not titrated it but we have used doses down to $6 \times 10^3$ with complete success ; $5 \times 10^4$ cells was chosen as a big dose and was fatal in unprotected C3H/C57 $F_1$ mice in under 20 days. We always challenged with freshly isolated cells to avoid the uncertainties involved in using cells stored in liquid nitrogen. However, both the tumour and the inbred mice can of course vary during repeated passage. Whenever possible the lymphocytes were isolated 7 days after the last immunising dose since lymphocyte immune-activity is maximal at this time whereas humoral-antibody titres are still low (Brondz, 1964). This was the reason for our schedule of repeated injections in preparing the C57 lymphocyte donors. In order to avoid damaging their antigenic structure, BP8 cells were killed by freeze/thawing, and *intimate mixing* with Freund's adjuvant was achieved without heat development by the emulsification techniques we employed. Some difficulty was experienced in isolating lymphocytes from the small quantity of blood available from mice. Care was taken to use non-wettable plastic or siliconed-glass apparatus. Viable counts showed good recovery of lymphocytes, practically free from other white cells, but with some red cell contamination.

The mixing of living BP8 cells with lymphocyte preparations just before injection i.p. appeared to have no deleterious effect on the BP8 cells, since the challenged animals invariably died, unless the lymphocytes were from allogeneic, and adequately immunised donors. Also " competent " lymphocytes gave protection when injected 24 hours after the tumour dose. Kidd and Toolan (1950) found a marked inhibition of tumour growth when susceptible mice were injected with pre-incubated mixtures of lymphoma cells and minced lymph nodes from mice with regressed lymphoma. However, Winn (1961), using sarcoma (SaI), found that incubation of cell mixtures before injection into host mice had no effect on the level of activity observed and concluded that the two cell types did not react *in vitro*. Indeed, the results obtained by Kidd and Toolan (1950) could have been due to the activity of the lymphocytes *in vivo*.

The spleen and lymph node hash used in our preliminary experiments would have contained other cells besides lymphocytes. Bennett (1965) has shown specific suppression of tumour growth by isolated peritoneal macrophages from immunised mice, but these were less effective than the lymphocytes from the same peritoneal exudates. Also Gough, Elves and Israels (1965) have shown that macrophages can develop from lymphocytes *in vitro* (in the presence of polymorphs). Our experiments indicated that blood lymphocyte preparations had most anti-tumour activity, on a per-cell basis, and the number of macrophages in these preparations must have been very small. The evidence in favour of the lymphocyte being the cell involved, accumulated since the pioneer work of Landsteiner and Chase (1942), Billingham, Brent and Medawar (1954) and Mitchison (1955) now appears very strong.

Certain alternative explanations of the destruction of tumour cells by " competent " lymphocytes seem to be eliminated by the experiments described in Part 3 of the results.

(i) There is no evidence of any non-specific activity of allogeneic lymphocytes from non-immunised donor C57 mice against BP8 cells. We found no sign of runt disease in any long-term survivors, so we have no indication that allogeneic C57 donor lymphocytes acted against the host C3H/C57 $F_1$ or C3H mice.

(ii) Bubenik and Koldovsky (1964) have shown that anti-tumour immunity can be transferred with serum, and Stück, Boyse, Old and Carswell (1964) found cytophilic antibody present in several types of mouse leukaemia. However, we do not believe that humoral antibody or cytophilic antibody (Boyden, 1963) can explain the antitumour activity of our lymphocyte preparations because they were all well washed in TC 199 and the only possible transfer of $\gamma$ globulin would be that absorbed on to any red cells present. Also in experiment 6 (ii), 1 ml. of serum from mice (whose lymphocytes gave good protection) gave no protection when incubated with normal lymphocytes and the combination was injected i.p. The findings of Algire, Weaver and Prehn (1957), that homografts in millipore diffusion chambers survived if no cells could get through the pores, also discount the role of cytophilic antibody.

(iii) We obtained definite evidence (Experiments 1 and 5) that lymphocytes from C57 mice treated with BP8 cells plus Freund's adjuvant gave better antitumour protection than lymphocytes from mice immunised with BP8 cells alone. However, lymphocytes from mice injected with Freund's adjuvant alone (Experiment 6, iii) failed to give any long-term survivors. Thus Freund's adjuvant alone did not produce any useful non-specific enhancement of lymphocyte activity against H2 histocompatibility antigens on the BP8 cells.

(iv) It also seemed unlikely that BP8 cells were simply serving as a source of H2 histocompatibility antigens (Haughton, 1964), since lymphocytes could not be sensitised by large doses of C3H spleen and liver cells plus Freund's adjuvant (Experiment 7). It is possible that we failed to sensitise the C57 mice to C3H

spleen and liver cells even though we used large doses and these cells are known to " abound " in most of the important H2 histocompatibility antigens. It seems more likely that the BP8 cells have a tumour-specific antigen.

(v) (vi) (vii) (viii). Foley (1953), Prehn and Main (1957), Prehn (1960), Klein and Sjögren (1960), Tuffrey and Batchelor (1964), Möller (1964) and others have shown that chemically induced mouse tumours were capable of immunising isogeneic mice. Zilber and Gamaleya (1957), using anaphylactic reactions, found evidence of tumour-specific antigens (see also Old and Boyse, 1964, for a review of virus-induced tumours and specific antigens). If BP8 has a tumour-specific antigen then it should be possible to immunise C3H mice against it. We fail to show protection with lymphocytes from C3H donors which had been immunised with BP8 cells plus Freund's adjuvant (Experiment 8) ; and our first attempt to immunise C3H mice against direct challenge with living BP8 cells also failed (Experiment 9). However, the results of Experiment 10 were in favour of a tumour-specific antigen, since C3H mice injected with BP8 cells plus Freund's adjuvant could resist challenge with living BP8 cells if the formation of ascitic fluid was controlled by restriction of fluid intake. However, the tumour had been passaged for nearly 9 months and changes in the histocompatibility antigens could have occurred in tumour and host.

We think the activity of the sensitised small lymphocytes against the tumour cell merits further study, in particular with regard to the mechanisms by which the lymphocyte damages the tumour cell. With more knowledge it might be possible to use allogeneic, anti-tumour competent lymphocytes as a therapeutic agent in human cancer.

### SUMMARY

C3H/C57 $F_1$ mice or C3H mice were protected by lymphocytes taken from C57 mice (injected with freeze/thawed BP8 cells plus Freund's complete adjuvant) against fatal challenge by living BP8 cells given i..p

These lymphocytes would also protect if injected i.p. 24 hours after the tumour cells.

In control experiments no protection was given by lymphocytes taken from non-immunised C57 mice, or those injected with C3H liver and spleen cells plus adjuvant, or adjuvant alone.

Some protection against challenge with BP8 cells was seen in C3H mice pre-immunised with BP8 cells and Freund's adjuvant, but only when fluid intake was restricted about the time of the onset of ascites.

We wish to thank Professor R. R. A. Coombs for help and advice, and Dr. L. B. Fraser for help in preparing the manuscript.

### REFERENCES

ALGIRE, G. H., WEAVER, J. M. AND PREHN, R. T.—(1957) *Ann. N.Y. Acad. Sci.*, **64**, 1009.

BENNETT, B.—(1965) *J. Immun.*, **95**, 656.

BILLINGHAM, R. E., BRENT, L. AND MEDAWAR, P. B.—(1954) *Proc. R. Soc.* B., **143**, 58.

BOYDEN, S. V.—(1963) *Ciba Fdn. Symp., on Transplantation*, edited by A. E. W. Wolstenholme and M. P. Cameron, London (Churchill) p. 6.

Brondz, B. D.—(1964) *Folia biol., Praha*, **10**, 164.
Bubenik, J. and Koldovsky, P.—(1964) *Folia biol., Praha*, **10**, 427.
Foley, E. J.—(1953) *Cancer Res.*, **13**, 835.
Gough, J., Elves, M. W. and Israels, M. C. G.—(1965) *exp. Cell Res.*, **38**, 476.
Gowans, J. L.—(1965) *Br. med. Bull.*, **21**, 106.
Haughton, G.—(1964) *Transplantation*, **2**, 251.
Janowsky, D. S., Rosenau, W. and Moon, H. D.—(1964) *Proc. Soc. exp. Biol. Med.*, **115**, 77.
Kidd, J. G. and Toolan, H. W.—(1950) *Fedn. Proc. Fedn. Am. Socs. exp. Biol.*, **9**, 385.
Klein, E. and Sjögren, H. O.—(1960) *Cancer Res.*, **20**, 452.
Landsteiner, K. and Chase, M. W.—(1942) *Proc. Soc. exp. Biol. Med.*, **49**, 688.
Medawar, P. B.—(1963) *Ciba Fdn. Study Grps.*, No. 16, ‘The immunological competent cell ; its nature and origin’ edited by G. E. W. Wolstenholme and J. Knight, London (Churchill) p. 110.
Mitchison, N. A.—(1955) *J. exp. Med.*, **102**, 157.
Möller, G.—(1964) *Nature, Lond.*, **204**, 846.
Old, J. L. and Boyse, E. A.—(1964) *A. Rev. Med.*, **15**, 167.
Prehn, R. T.—(1960) *Cancer Res.*, **20**, 1614.
Prehn, R. T. and Main, J. M.—(1957) *J. natn. Cancer Inst.*, **18**, 769.
Skoog, W. A. and Beck, W. S.—(1956) *Blood*, **11**, 436.
Stück, B., Boyse, E. A., Old, L. J. and Carswell, E. A.—(1964) *Nature Lond.*, **203**, 1033.
Tuffrey, M. A. and Batchelor, J. R.—(1964) *Nature Lond.*, **204**, 349.
Winn, H. J.—(1961) *J. Immun.*, **86**, 228.
Zilber, L. A. and Gamaleya, N. F.—(1957) *J. natn. Cancer Inst.*, **18**, 341.